# Low serum dehydroepiandrosterone is associated with diabetic dyslipidemia risk in males with type 2 diabetes

**DOI:** 10.3389/fendo.2023.1272797

**Published:** 2023-11-24

**Authors:** Shanshan Chen, Shuo Li, Xinxin Zhang, Yuxin Fan, Ming Liu

**Affiliations:** Department of Endocrinology and Metabolism, Tianjin Medical University General Hospital, Tianjin, China

**Keywords:** dehydroepiandrosterone, dehydroepiandrosterone sulfate, dyslipidemia, diabetes mellitus, type 2, androgen

## Abstract

**Objective:**

Sex steroid hormones are associated with the advancement of metabolic diseases such as dyslipidemia. This cross-sectional study aimed to investigate the relationship between dehydroepiandrosterone, dehydroepiandrosterone sulfate, androstenedione, and testosterone levels and the risk of dyslipidemia in people with type 2 diabetes mellitus.

**Materials and Methods:**

The analysis included 1,927 patients with type 2 diabetes mellitus. Serum dehydroepiandrosterone, dehydroepiandrosterone sulfate, androstenedione, and testosterone levels were determined using lipid chromatography-tandem mass spectrometry. Multivariable analyses were performed to investigate the association between the variables and dyslipidemia.

**Results:**

The multivariable-adjusted odds ratio (OR) and 95% confidence interval (CI) of dyslipidemia across DHEA tertiles were 0.39 and 0.24-0.64, respectively (p trend = 0.001). This relationship was still maintained when analyzed as a continuous variable (odds ratio, 0.96; 95% confidence interval, 0.92–0.99; P < 0.01). However, in males with type 2 diabetes mellitus, no significant correlations were found between rising levels of dehydroepiandrosterone sulfate, androstenedione, and total testosterone and the risk of dyslipidemia (all P > 0.05). Furthermore, there was no significant association between androgen precursors and total testosterone with regard to the risk of developing dyslipidemia (all P > 0.05).

**Conclusions:**

Serum dehydroepiandrosterone levels were substantially and adversely correlated with dyslipidemia in adult men with T2DM. These results indicated that dehydroepiandrosterone may have an essential role in the development of dyslipidemia. More prospective research is required to validate this link.

## Introduction

Dyslipidemia is a prevalent problem in people with type 2 diabetes mellitus (T2DM), presenting with varied manifestations depending on the amount and/or quality of lipoproteins. Primary and secondary dyslipidemia, which have strictly inherited origins, and dyslipidemia caused by other diseases or pathological conditions are frequently distinguished depending on the etiology. Genetic mutations are the most common cause of primary dyslipidemia. Secondary dyslipidemia is caused by other conditions (such as diabetes or obesity), with the latter being more common (1, 2). Patients with T2DM are more likely to develop coronary artery disease as a result of a type of secondary dyslipidemia known as diabetic dyslipidemia (3). Cardiovascular disease is the leading cause of morbidity and mortality in patients with diabetes, and it is highly influenced by dyslipidemia (4). Diabetic dyslipidemia is characterized by increasing low-density lipoprotein cholesterol (LDL-C) and triglyceride (TG) levels and decreased high-density lipoprotein cholesterol (HDL-C) levels (5).

Dehydroepiandrosterone (DHEA) and its sulfate ester dehydroepiandrosterone sulfate (DHEAS) are precursors of sex hormones secreted by the adrenal cortex (6, 7). In particular, DHEA is produced by the adrenocorticotropic hormone (ACTH) reaction and is converted into androstenedione, which is metabolized in the liver by dehydrogenation to testosterone or aromatization to estrogen (8). In the human body, the two most abundant cyclic steroid hormones are DHEA and DHEAS (9). DHEA and DHEAS blood concentrations in men and women reduce with age (10, 11). DHEA has been found to improve diabetes and obesity in animal models. DHEA may alleviate diabetes by increasing pancreatic insulin output as well as insulin sensitivity in the liver, fat, and muscle (12–14). Furthermore, by decreasing lipid synthesis and raising resting metabolic rate and calorie output, DHEA may have anti-obesity benefits (15, 16). However, the connection between DHEA and dyslipidemia remains unclear.

The purpose of this cross-sectional study was to investigate the link between DHEA, DHEAS, testosterone, and androstenedione levels and the risk of dyslipidemia in T2DM patients.

## Materials and methods

### Study participants

**Figure 1:**
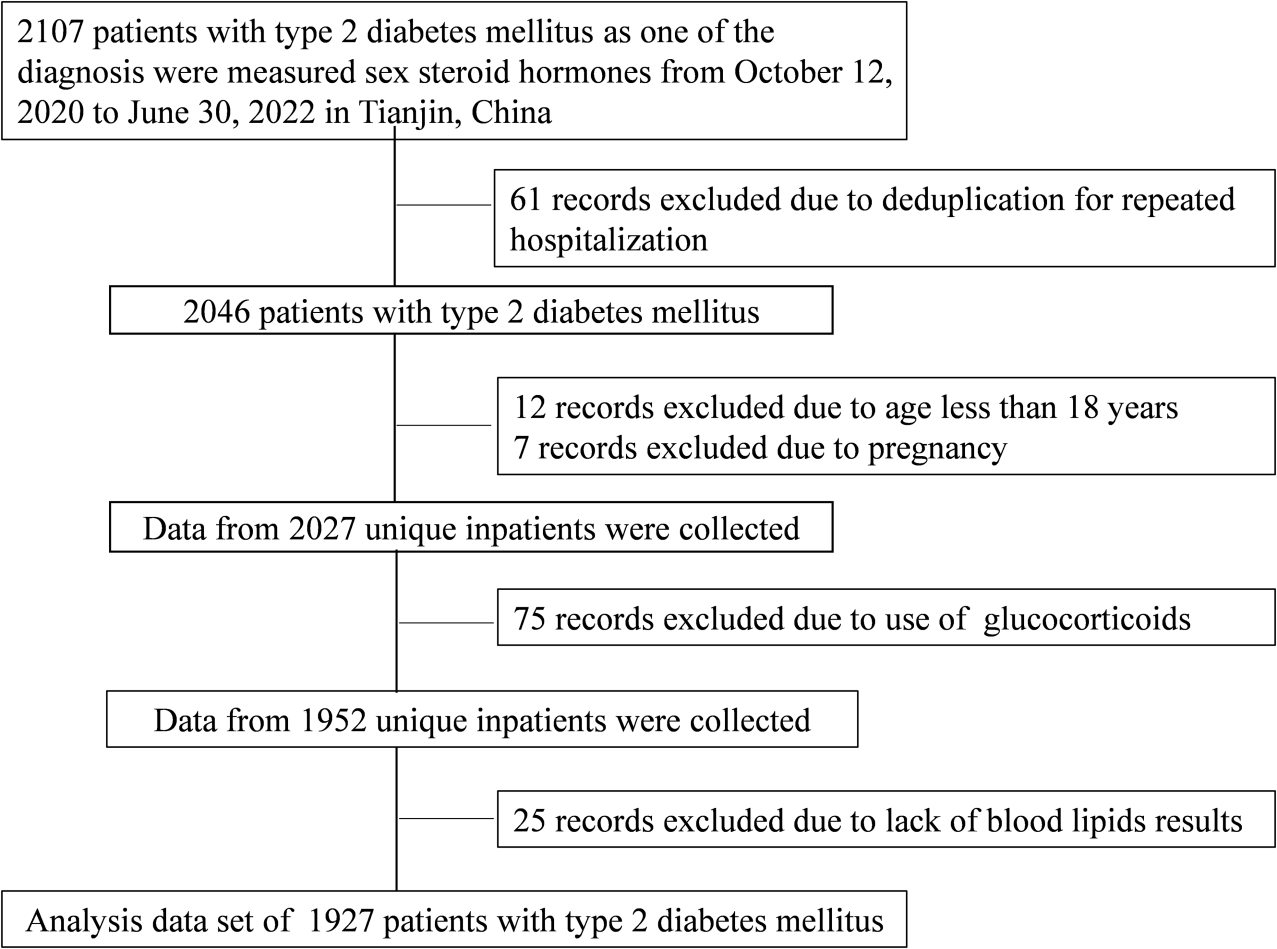
Flow chart of identification of study population. A total of 1,927 patients with T2DM were included in the final analysis based on the exclusion criteria. T2DM, type 2 diabetes mellitus.

Medical records from 2,107 T2DM patients were evaluated between October 12, 2020, and June 30, 2022. All patients were treated at Tianjin Medical University General Hospital’s Department of Endocrinology and Metabolism, and their sex steroids hormone levels were assessed. When a person had numerous hospital records, only one of them was chosen. Patients under the age of 18 who did not have lipid data and pregnant women were excluded from the trial. Given that hormone usage can impact sex steroid hormone levels, this was also an exclusion criterion. **Figure 1** depicts the patient-identifying method. Based on exclusion criteria, 1,927 T2DM individuals were enrolled in the final investigation.

The Tianjin Medical University General Hospital Institutional Review Board approved the project. The informed consent requirement (approval number: IRB2020-YX-027-01) was waived as the patient data were collected from the Department of Endocrinology and Metabolism’s electronic medical record and the patient’s identity was obscured.

### Covariates

Age, sex, lifestyle factors, insurance, history of diabetes, hypertension, and dyslipidemia, height and weight, systolic and diastolic blood pressures (SBP, DBP), as well as blood levels for glycated hemoglobin (HbA1c), total cholesterol (TC), TG, LDL-C, HDL-C, uric acid (UA), ACTH, and C-reactive protein (CRP), were all taken from medical records. Body mass index (BMI) is computed by dividing a patient’s weight in kilograms by their height in meters squared. Cigarette smoking was defined as smoking more than one cigarette per day for at least one year, and participants were divided into three groups: never-smokers, prior smokers (those who had quit smoking for at least 6 months), and current smokers. Alcohol use was defined as consuming more than 400 mL alcohol/week > 1 year. Participants were divided into three groups: non-drinkers, former drinkers (characterized by temperance for at least 6 months), and current drinkers. The Endocrine and Metabolism Laboratory at Tianjin Medical University’s General Hospital used liquid chromatography-tandem mass spectrometry to evaluate the levels of androgen precursors. After the patients had fasted for at least 8 hours, venous blood samples were taken at 6 a.m. on the second day of admission. Our lab performed the steroid hormone assay as previously described (17–19).

### Definitions

A fasting blood glucose (FBG) level of 7.0 mmol/L, a 2-hour plasma glucose level of 11.1 mmol/L, an HbA1c level of 6.5%, a self-identified diagnosis of diabetes, or usage of hypoglycemic drugs was considered diabetes (20). SBP of 140 mmHg, DBP of 90 mmHg, self-identified diagnosis of hypertension, or usage of antihypertensive medication were all considered hypertension (21). Dyslipidemia was defined as TC >6.20 mmol/L, LDL-C >4.13 mmol/L, TG >2.25 mmol/L or HDL-C <1.03 mmol/L, or usage of lipid-lowering drugs (22).

### Statistical analyses

For continuous variables, standard deviations (SDs) and means are used to express normally distributed data, and the Student’s t-test was used to compare the groups. Interquartile ranges (IQRs) and medians were employed to represent non-normally distributed data, and the Mann–Whitney U test was employed to compare the groups. The chi-square test was used to compare results between the groups for categorical variables, which are expressed as numbers and frequencies.

The value and significance of the correlation coefficients of sex hormone concentrations in males and females were estimated using the Spearman’s correlations. To explore the influence of sex hormones on dyslipidemia, three models were adjusted for covariates. Model 1 was corrected for age; Model 2 was further adjusted for current alcohol and smoking use; and Model 3 was corrected for Model 2 plus BMI, type of insurance, T2DM duration, SBP, ACTH, and HbA1c. Testosterone, androstenedione, DHEAS, and DHEA levels were individually divided into tertile groups. The lowest tertile was utilized as the reference group for binary logistic regression analysis. After adjusting for age, BMI, current smoking, insurance type, current alcohol consumption, T2DM duration, SBP, and HbA1c, the dose–response relationship between sex hormones and dyslipidemia was analyzed with restrictive cubic splines. A p-value < 0.05 (two-tailed test) was considered significant for correlation intervals (CIs). IBM SPSS Statistics for Windows, version 25.0 (IBM Corporation, Armonk, NY, USA) was used for all analyses.

## Results

### Characteristics of the study participants

In the study’s male cohort of 1021, 786 (77%) of the participants had dyslipidemia. **Table 1** shows that males with dyslipidemia had higher BMI, TG, FBG, UA, CRP, and creatinine (Cr) values, as well as a higher prevalence of hypertension than those in males with no dyslipidemia. However, the mean age, HDL-C, and duration of T2DM among males with dyslipidemia were lower than those in males with non-dyslipidemia (P < 0.05). In males with dyslipidemia, total testosterone (TT) and DHEA levels were decreased (P < 0.05).

**Table 1:** Demographic characteristics and clinical parameters of patients with and without dyslipidemia (men, n=1021).

	Men (n=1021)		
	Non-Dyslipidemia	Dyslipidemia	P
Participants, %	235 (23.0)	786 (77.0)	–
Age, years	57.54±13.95	54.40±14.55	0.004**
BMI, Kg/m2	26.10±4.90	27.67±5.26	<0.0001****
Current smoking, %	99 (42.1)	373 (47.5)	0.135
Current drinking, %	109 (46.4)	354 (45.0)	0.783
Insurance type, %			
Urban workers	197 (83.8)	661 (84.1)	
Non-working urban residents	28 (11.9)	76 (9.7)	
Self-pay	10 (4.3)	49 (6.2)	
Duration of type 2 diabetes mellitus, year	8.50 (2.00-15.00)	7.00 (0.71-14.00)	0.018*
Hypertension, %	124 (52.8)	501 (63.7)	0.002**
Blood pressure, mmHg			
Systolic	137.62±17.91	137.63±18.90	0.676
Diastolic	83.97±12.19	84.92±12.29	0.259
TC, mmol/L	4.68±0.79	4.89±1.79	0.837
TG, mmol/L	1.30 (0.96-1.64)	2.05 (1.37-3.02)	<0.001***
LDL-C, mmol/L	2.92±0.70	2.94±1.03	0.784
HDL-C, mmol/L	1.22±0.21	0.97±0.23	<0.001***
FBG, mmol/L	7.2±2.62	7.84±3.01	0.014*
HbA1c,%	8.51±2.38	8.71±2.23	0.085
UA, μmol/L	329.41±92.65	369.53±107.76	<0.001***
CRP, mg/dl	0.23 (0.15-0.35)	0.28 (0.17-0.56)	<0.001***
Cr, μmol/L	63.00 (53.00-73.00)	65.00 (55.00-78.00)	0.008**
TT, nmol/L	13.34 (9.49-18.17)	11.86 (9.12-15.07)	<0.001***
Androstenedione, nmol/L	2.06 (1.55-2.63)	1.98 (1.48-2.57)	0.288
DHEA, nmol/L	8.51 (6.25-13.23)	8.21 (5.42-12.06)	0.050*
DHEAS, μmol/L	4.07 (2.38-5.73)	3.91 (2.43-5.93)	0.592
ACTH, pg/mL	35 (25.00-49.95)	32.3 (22.4-47.90)	0.145
Lipid lowing, n (%)		260 (33.2)	

* P < 0.05, ** P < 0.01, *** P < 0.001, **** P < 0.0001.

BMI, body mass index; T2DM, type 2 diabetes mellitus; TC, total cholesterol; TG, triglycerides; HDL-C, high-density lipoprotein cholesterol; LDL-C, low-density lipoprotein cholesterol; UA, uric acid; CRP, C-reactive protein; HbA1c, glycosylated hemoglobin; TT, total testosterone; DHEA, dehydroepiandrosterone; DHEAS, dehydroepiandrosterone sulfate; ACTH, adrenocorticotropic hormone.

Bold results are statistically significant.

A total of 633 (69.9%) females met the criteria for dyslipidemia. Females with dyslipidemia had higher TC, TG, FBG, HbA1c, UA, and CRP levels but lower mean HDL-C levels than those of females without dyslipidemia (all P < 0.05). Similar to that of males, females with dyslipidemia had a higher prevalence of hypertension (all P < 0.05). Lower DHEA levels in females with dyslipidemia were also observed (P = 0.008; **Table 2**).

**Table 2:** Demographic characteristics and clinical parameters of patients with and without dyslipidemia (women, n=906).

	Women (n=906)		
	Non-Dyslipidemia	Dyslipidemia	P
Participants, %	273(30.1)	633(69.9)	-
Age, years	58.27±14.63	56.71±14.98	0.225
BMI, Kg/m2	26.99±6.3	27.53±5.82	0.059
Current smoking, %	10(3.7)	38(6.1)	0.144
Current drinking, %	6(2.2)	24(3.8)	0.213
Insurance type, %			
Urban workers	228(83.5)	516(81.5)	
Non-working urban residents	29(10.6)	90(14.2)	
Self-pay	16(5.9)	27(4.3)	
Duration of type 2 diabetes mellitus, year	7.00 (0.50-13.00)	7.00 (1.00-16.00)	0.205
Hypertension, %	273	633	0.030*
Blood pressure, mmHg			
Systolic	136.67±19.52	135.75±11.79	0.743
Diastolic	80.64±11.28	80.83±11.58	0.767
TC, mmol/L	4.81±0.7	5.13±1.52	0.032*
TG, mmol/L	1.33 (1.04-1.63)	1.95 (1.47-2.82)	<0.001***
LDL-C, mmol/L	2.88±0.64	3.11±1.1	0.075
HDL-C, mmol/L	1.3±0.22	1.05±0.26	<0.001***
FBG, mmol/L	7.09±2.45	7.89±2.99	<0.0001****
HbA1c,%	8.06±1.93	8.63±2.13	<0.001***
UA, μmol/L	296.37±93.76	334.13±105.87	<0.001***
CRP, mg/dl	0.27 (0.16-0.46)	0.35 (0.19-0.60)	<0.001***
Cr, μmol/L	46.00 (40.00-55.00)	47.00 (39.00-58.00)	0.383
TT, nmol/L	0.50 (0.35-0.76)	0.48 (0.32-0.72)	0.129
Androstenedione, nmol/L	1.74 (1.18-2.61)	1.78 (1.15-2.51)	0.918
DHEA, nmol/L	9.25 (5.93-14.06)	8.13 (5.01-12.62)	0.008**
DHEAS, μmol/L	2.20 (1.44-3.64)	2.33 (1.32-3.82)	0.968
ACTH, pg/mL	29.4 (18.70-43.00)	28.4 (19.63-43.18)	0.9160
Lipid lowing, n (%)		234(37.1)	

* P < 0.05, ** P < 0.01, *** P < 0.001, **** P < 0.0001.

BMI, body mass index; T2DM, type 2 diabetes mellitus; TC, total cholesterol; TG, triglycerides; HDL-C, high-density lipoprotein cholesterol; LDL-C, low-density lipoprotein cholesterol; UA, uric acid; CRP, C-reactive protein; HbA1c, glycosylated hemoglobin; TT, total testosterone; DHEA, dehydroepiandrosterone; DHEAS, dehydroepiandrosterone sulfate; ACTH, adrenocorticotropic hormone.

Bold results are statistically significant.

### Prevalence of dyslipidemia by tertiles of TT, androstenedione, DHEA, and DHEAS levels

**Figure 2:**
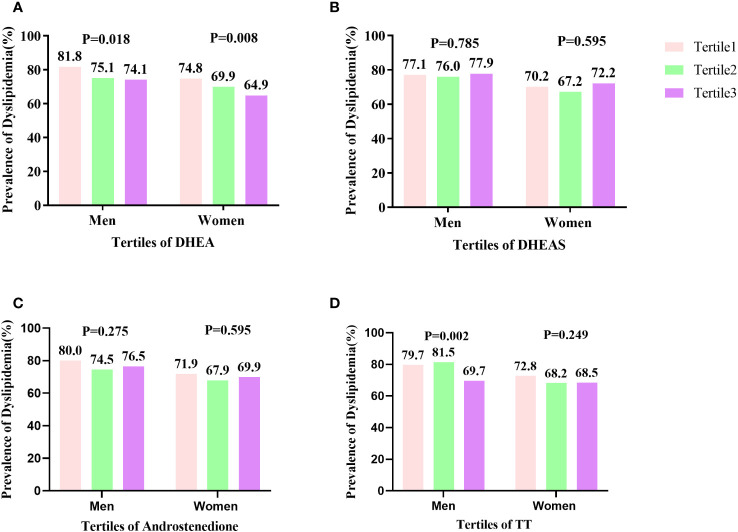
Prevalence of dyslipidemia by tertiles of TT, androstenedione, DHEA, and DHEAS levels: **(A)** prevalence of dyslipidemia by DHEA level; **(B)** prevalence of dyslipidemia by DHEAS level; **(C)** prevalence of dyslipidemia by androstenedione level; **(D)** prevalence of dyslipidemia by TT level. The prevalence of dyslipidemia significantly decreased in line with increasing tertiles of serum DHEA levels in females (P = 0.008) and (P = 0.018) in males. The percentage of males with dyslipidemia significantly decreased in accordance with increasing tertiles of serum TT level (P = 0.002).

**Figure 2** shows the prevalence of dyslipidemia assessed in males and females in tertiles of serum DHEA, DHEAS, TT, and androstenedione levels. As the serum DHEA level of tertile increased, the percentage of males with dyslipidemia reduced considerably, with 81.8%, 75.1%, and 74.1% in tertiles 1–3, respectively (P < 0.05). Moreover, in males, the prevalence of dyslipidemia rose with increasing TT level tertiles, with 79.7%, 74.5%, and 69.7% in tertiles 1–3, respectively (P < 0.05). Dyslipidemia was more common in females as DHEA level tertiles increased, with 74.8%, 69.9%, and 64.9% in tertiles 1–3, respectively (P < 0.05).

### Relationships between DHEA, DHEAS, androstenedione, and TT levels with dyslipidemia risk

**Table 3** shows the associations between the risk of dyslipidemia in males after correcting for DHEA, androstenedione, DHEAS, and testosterone levels, as well as confounding factors, in logistic regression analysis. After adjusting for relevant variables (age, current smoking, BMI, current drinking, T2DM duration, insurance type, SBP, ACTH, and HbA1c) in model 3, the risk of dyslipidemia was reduced with increasing serum DHEA levels (OR, 0.39, tertile 3 *vs*. tertile 1; 95% CI, 0.24–0.64, P < 0.001 for trend). Moreover, when DHEA level was measured as a continuous variable, each SD increase was related to a decrease of 4% in the odds of developing dyslipidemia in model 3 (OR, 0.96; 95% CI, 0.92-0.99; P = 0.009). Higher concentrations of serum androstenedione, TT, and DHEAS were not correlated with a higher risk of dyslipidemia (all P > 0.05).

**Table 3:** Odds ratios of dyslipidemia among men by different types of androgens.

	Odds ratios (95% CI)		
	Model1	Model2	Model3
DHEA			
Tertile1	1	1	1
Tertile2	0.55 (0.38,0.81)	0.56 (0.38,0.83)	0.57 (0.37-0.88)
Tertile3	0.40 (0.26,0.61)	0.41 (0.27,0.62)	0.39 (0.24-0.64)
P for trend	<0.001***	<0.001***	<0.001***
Per SD increment	0.95 (0.93,0.98)	0.96 (0.93,0.99)	0.96 (0.92-0.99)
P value	<0.001***	0.003**	0.009**
DHEAS			
Tertile1	1	1	1
Tertile2	0.82 (0.57,1.19)	0.83 (0.58,1.20)	0.95 (0.63-1.43)
Tertile3	0.79 (0.53,1.18)	0.81 (0.54,1.21)	0.82 (0.51-1.30)
P for trend	0.454	0.517	0.676
Per SD increment	0.98 (0.92,1.04)	1.00 (0.93,1.06)	0.99 (0.92-1.07)
P value	0.476	0.877	0.798
Androstenedione			
Tertile1	1	1	1
Tertile2	0.68 (0.47,0.98)	0.69 (0.48,0.99)	0.65 (0.43-0.98)
Tertile3	0.73 (0.51,1.06)	0.75 (0.52,1.09)	0.81 (0.5201.26)
P for trend	0.098	0.12	0.117
Per SD increment	0.89 (0.77,1.04)	0.90 (0.77,1.05)	0.96 (0.79-1.17)
P value	0.133	0.167	0.689
TT			
Tertile1	1	1	1
Tertile2	1.1 (0.78,1.66)	1.11 (0.75,1.63)	1.42(0.92-2.18)
Tertile3	0.60 (0.42,0.86)	0.61 (0.43,0.87)	0.84(0.56-1.27)
P for trend	<0.001***	0.002**	0.040*
Per SD increment	0.95 (0.92,0.97)	0.95 (0.92,0.97)	0.97(0.94-1.00)
P value	<0.001***	<0.001***	0.089

* P < 0.05, ** P < 0.01, *** P < 0.001.

Model 1: adjusts for age.

Model2: model1+current smoking (yes/no), current drinking (yes/no).

Model 3: model2+BMI, insurance type, duration of diabetes, SBP, ACTH, and HbA1c.

BMI, body mass index; T2DM, type 2 diabetes mellitus; SBP, systolic blood pressure; ACTH, adrenocorticotropic hormone; HbA1c, glycosylated hemoglobin; TT, total testosterone; DHEA, dehydroepiandrosterone; AE, androstenedione; DHEAS, dehydroepiandrosterone sulfate; CI confidence interval; SD, standard deviation.

Bold results are statistically significant.

**Table 4** illustrates that lower serum DHEA levels in females with T2DM were related to a higher OR of dyslipidemia in Models 1 (OR, 0.50; 95% CI, 0.34–0.73; P < 0.05) and 2 (OR, 0.49; 95% CI, 0.33–0.73; P < 0.05). When DHEA level was analyzed as a continuous variable, a 1-SD increase in serum DHEA level was associated with a 3% reduction in dyslipidemia risk in females in Models 1 (OR, 0.97; 95% CI, 0.95–0.99; P = 0.005) and 2 (OR, 0.97; 95% CI, 0.95–0.99; P = 0.003). In the fully corrected mode, however, this link was not statistically significant (OR, 0.98; 95% CI, 0.96–1.00; P > 0.05). There were no statistically significant connections between higher DHEA, androstenedione, and TT levels and the hazard ratio of dyslipidemia. (P > 0.05).

**Table 4:** Odds ratios of dyslipidemia among women by different types of androgens.

	Odds ratios (95% CI)		
	Model1	Model2	Model3
DHEA			
Tertile1	1	1	1
Tertile2	0.72(0.50,1.03)	0.70(0.48,1.01)	0.69(0.46-1.04)
Tertile3	0.50(0.34,0.73)	0.49(0.33,0.73)	0.56(0.36-0.88)
P for trend	0.002**	0.002**	0.036*
Per SD increment	0.97(0.95,0.99)	0.97(0.95,0.99)	0.98(0.96-1.00)
P value	0.005**	0.003**	0.073
DHEAS			
Tertile1	1	1	1
Tertile2	0.84(0.59,1.19)	0.83(0.59,1.18)	0.92(0.6201.37)
Tertile3	1.00(0.68,1.46)	0.98(0.66,1.44)	1.15(0.74-1.78)
P for trend	0.524	0.535	0.5940
Per SD increment	0.96(0.88,1.04)	0.95(0.88,1.04)	0.96(0.88-1.06)
P value	0.316	0.264	0.4440
Androstenedione			
Tertile1	1	1	1
Tertile2	0.79(0.56,1.13)	0.82(0.57,1.16)	0.78(0.52-1.15)
Tertile3	0.79(0.54,1.16)	0.77(0.53,1.14)	0.95(0.60-1.51)
P for trend	0.359	0.376	0.3940
Per SD increment	0.99(0.88,1.12)	0.99(0.88,1.11)	1.07(0.93-1.24)
P value	0.875	0.819	0.3280
TT			
Tertile1	1	1	1
Tertile2	0.79(0.56,1.12)	0.81(0.57,1.16)	0.86(0.58-1.29)
Tertile3	0.77(0.54,1.10)	0.77(0.54,1.10)	0.80-0.54-1.20)
P for trend	0.284	0.314	0.557
Per SD increment	0.77(0.56,1.05)	0.77(0.56,1.06)	0.85(0.55-1.31)
P value	0.102	0.106	0.46

* P < 0.05, ** P < 0.01.

Model 1: adjusts for age.

Model2: model1+current smoking (yes/no), current drinking (yes/no).

Model 3: model2+BMI, insurance type, duration of diabetes, SBP, ACTH, and HbA1c.

BMI, body mass index; T2DM, type 2 diabetes mellitus; SBP, systolic blood pressure; ACTH, adrenocorticotropic hormone; HbA1c, glycosylated hemoglobin; TT, total testosterone; DHEA, dehydroepiandrosterone; AE, androstenedione; DHEAS, dehydroepiandrosterone sulfate; CI confidence interval; SD, standard deviation.

Bold results are statistically significant.

## Discussion

In this cross-sectional study, we present evidence for the connection of DHEA with the risk of developing dyslipidemia in T2DM patients. After correcting for potential confounders such as age, smoking, alcohol use, BMI, T2DM duration, insurance type, SBP, ACTH, and HbA1c levels, we found that lowered serum DHEA levels in males with T2DM are independently associated with the risk of dyslipidemia. In females with T2DM, DHEA levels and the risk of dyslipidemia had no statistically significant connection. Moreover, there was little association between androstenedione, TT, and DHEA levels and the incidence of dyslipidemia in males and females with T2DM.

DHEA secretion in humans is age-related (23). DHEA is the primary byproduct of prenatal adrenal production, resulting in high circulating DHEA levels at birth. Throughout the primary year of life, serum DHEA concentrations fall to nearly inconspicuous levels, which corresponds to the postnatal development of the fetal adrenal gland. DHEAS levels continue little until the sixth to the tenth year of life when they gradually increase due to increasing DHEA secretion from the adrenal reticular zone, a condition known as adrenal primordium (24, 25).

DHEA has been demonstrated to have a positive effect on the progression of T2DM. Yamaguchi et al. discovered that serum DHEA levels were considerably less in patients with T2DM having high blood insulin levels compared with those in controls and those with T2DM without high serum insulin levels (26). Previous research has linked raised serum DHEA levels to an elevated risk of coronary heart disease (CHD) in men. Lower blood DHEA concentrations were connected with the occurrence of CHD, regardless of established risk factors, according to prospective research enrolling males between the ages of 40 and 70 (27). Low-dose DHEA treatment enhanced vascular endothelial functionality and insulin sensitivity while decreasing plasminogen activator inhibitor type 1 concentration in a randomized, double-blind investigation of men with hypercholesterolemia (28). In a 1-year randomized controlled trial, a healthy population aged 60-79 years was administered 50 mg/day of taking DHEA, and their serum DHEAS levels were restored to young adult levels after 6 months. DHEA treatment enhanced the density of bone minerals in women, primarily at the femoral neck, Ward’s triangle, and upper radius. In men, no effect on bone turnover was observed (29). According to a 4-month cross over controlled study involving patients with adrenal insufficiency randomized to receive DHEA or placebo, DHEA significantly reduced TC and HDL-C levels and increased overall well-being, compared to placebo (30). In addition, among 270 postmenopausal women who participated in the observational Women’s Ischemia Syndrome Evaluation (WISE) study, decreased DHEAS levels were found to be linked with a higher probability of cardiovascular disease and all-cause death (31).

However, findings regarding the link between DHEA and dyslipidemia are inconsistent. In a previous meta-analysis, DHEA supplementation was administered to both older men and postmenopausal women. DHEA supplementation decreased fat mass in older men (32). When compared to a placebo group, DHEA did not affect glycemia, insulin, or TC. This action depends on the conversion of DHEA metabolites into androgens or estrogens. In another study, DHEA treatment had no impact on TC, HDL-C, LDL, TG, serum glucose, weight, or BMI in postpartum women (33). In a crossover of double-blinding placebo-controlled research, DHEA supplementation had no consequence on serum insulin, glucose, or lipid profiles in aged men (34). DHEA treatment increased the concentrations of TC, phospholipids, LDL-C, TG, and apo-B in rabbits with hyperlipidemia fed with an atherogenic diet. DHEA, a weakened androgenic and probable predecessor of androgens and estrogens, might affect sex hormone levels as well as serum lipid concentrations indirectly by altering the hormone environment (35).

Findings from animal studies indicate that DHEA supplementation may be effective in reducing chronic complications of diabetes, however, the mechanism is unknown. The db/db mouse was used as a T2DM animal model. Coleman et al. discovered that DHEA therapy decreased hyperglycemia, increased insulin sensitivity, and retained islet function and β-cell structure in db/db mice (36). Kimura et al. found that DHEA reduced serum TNF levels in Zucker fatty rats (37), whereas Aoki et al. discovered that DHEA suppressed increased glucose-6-phosphatase and fructokinase levels in C57BL/Ksj-db/db mice (38). Campbell et al. DHEA activates the PI3-kinase/AKT pathway in the liver and the PI3K/atypical PKCzeta/lambda pathway in muscle (39). These researchers concluded that DHEA supplementation could help prevent insulin resistance. As a result, we hypothesized that DHEA’s alleviated insulin resistance effect explains certain aspects of the inverse connection between DHEA and diabetic dyslipidemia. Furthermore, a hepatic androgen receptor deficiency decreases fatty acid oxidation and increases hepatic *de novo* lipogenesis by decreasing PPARα expression, which causes hepatic steatosis and insulin resistance (40). This may also explain the link between low serum DHEA levels and dyslipidemia. The lack of a significant relationship between DHEA and dyslipidemia in women may be because of estrogenic protective mechanisms that improve lipid metabolism by preventing the accumulation of white adipose tissue by decreasing fatty acid and triglyceride production and lipogenesis (41).

There are some limitations to the current investigation. The association of DHEA, DHEAS, testosterone, and androstenedione with dyslipidemia does not establish a causal association due to the cross-sectional methodology. Second, we did not collect data on all the characteristics associated with dyslipidemia, such as eating habits, which may have limited our multivariable analysis. Third, the sample size was limited. As a result, future studies should rely on larger sample sizes. Fourth, because the study’s population was drawn from hospitalized patients, the results may not fully reflect the general community, which should be included as a control in future research. Finally, because this trial enrolled patients with T2DM, the results might not accurately reflect the general diabetic community.

As a result, after controlling for risk variables, lower serum DHEA levels in males with T2DM were found to be correlated with the risk of dyslipidemia. Interestingly, no associations were identified among DHEAS, androstenedione, testosterone, and the incidence of dyslipidemia in either males or females. According to our data, DHEA may play a part in the progression of dyslipidemia in males with T2DM. More prospective research is required to validate this link.

## Data availability statement

The raw data supporting the conclusions of this article will be made available by the authors, without undue reservation.

## Ethics statement

The studies involving humans were approved by The Tianjin Medical University General Hospital Institutional Review Board approval number: IRB2020-YX-027-01. The studies were conducted in accordance with the local legislation and institutional requirements. The ethics committee/institutional review board waived the requirement of written informed consent for participation from the participants or the participants’ legal guardians/next of kin because as the patient data were collected from the Department of Endocrinology and Metabolism’s electronic medical record and the patient’s identity was obscured. Written informed consent was obtained from the individual(s) for the publication of any potentially identifiable images or data included in this article.

## Author contributions

ML: Writing – review & editing. SC: Writing – original draft. SL: Data curation, Writing – review & editing. YF: Writing – review & editing. XZ: Writing – review & editing.

## References

[1] Kolovou PMKGD Anagnostopoulou KK Bilianou H Mikhailidis DP. Primary and secondary hypertriglyceridaemia. Curr Drug Targets (2009) 10(4):336–43. doi: 10.2174/138945009787846452 19355858

[2] Pirillo A Casula M Olmastroni E Norata GD Catapano AL. Global epidemiology of dyslipidaemias. Nat Rev Cardiol (2021) 18:689–700. doi: 10.1038/s41569-021-00541-4 33833450

[3] Scicali R Di Pino A Ferrara V Urbano F Piro S Rabuazzo AM. New treatment options for lipid-lowering therapy in subjects with type 2 diabetes. Acta Diabetol (2018) 55:209–18. doi: 10.1007/s00592-017-1089-4 29260404

[4] Nelson AJ Rochelau SK Nicholls SJ. Managing dyslipidemia in type 2 diabetes. Endocrinol Metab Clin North Am (2018) 47:153–73. doi: 10.1016/j.ecl.2017.10.004 29407049

[5] Wu L Parhofer KG. Diabetic dyslipidemia. Metabolism (2014) 63:1469–79. doi: 10.1016/j.metabol.2014.08.010 25242435

[6] Shackleton ERCHL Phillips A Chang T. Androstanediol and 5-androstenediol profiling for detecting exogenously administered dihydrotestosterone, epitestosterone, and dehydroepiandrosterone: potential use in gas chromatography isotope ratio mass spectrometry. Steroids (1997) 62(10):665–73. doi: 10.1016/S0039-128X(97)00065-2 9381514

[7] K.M. VA Eberling MD P. Physiological importance of dehydroepiandrosterone. Lancet (London England) (1994) 343(8911):1479–81. doi: 10.1016/S0140-6736(94)92587-9 7911183

[8] H.A. R Tait JF. *In vivo* conversion of dehydroisoandrosterone to plasma androstenedione and testosterone in man. J Clin Endocrinol Metab (1967) 27(1):79–88. doi: 10.1210/jcem-27-1-79 4225307

[9] Rutkowski K Sowa P Rutkowska-Talipska J Kuryliszyn-Moskal A Rutkowski R. Dehydroepiandrosterone (DHEA): hypes and hopes. Drugs (2014) 74:1195–207. doi: 10.1007/s40265-014-0259-8 25022952

[10] Kushnir MM Blamires T Rockwood AL Roberts WL Yue B Erdogan E. Liquid chromatography-tandem mass spectrometry assay for androstenedione, dehydroepiandrosterone, and testosterone with pediatric and adult reference intervals. Clin Chem (2010) 56:1138–47. doi: 10.1373/clinchem.2010.143222 20489135

[11] L. GA Liu CH Fischer UG Yen SSC. Marked attenuation of ultradian and circadian rhythms of dehydroepiandrosterone in postmenopausal women:evidence for a reduced 17,20-desmolase enzymatic activity. J Clin Endocrinol Metab (1990) 71:900–6. doi: 10.1210/jcem-71-4-900 2169480

[12] L. EH Coleman DL Applezweig N. Therapeutic effects of dehydroepiandrosterone metabolites in diabetes mutant mice (C57BL/ksJ-db/db). Endocrinology (1984) 115(1):239–43. doi: 10.1210/endo-115-1-239 6234160

[13] Aoki K Terauchi Y. Effect of dehydroepiandrosterone (DHEA) on diabetes mellitus and obesity. Vitam Horm (2018) 108:355–65. doi: 10.1016/bs.vh.2018.01.008 30029734

[14] S. RW Coleman DL Leiter EH. Effect of genetic background on the therapeutic effects of dehydroepiandrosterone (DHEA) in diabetes-obesity mutants and in aged normal mice. Diabetes (1984) 33(1):26–32. doi: 10.2337/diabetes.33.1.26 6228478

[15] Han DH Hansen PA Chen MM Holloszy JO. DHEA treatment reduces fat accumulation and protects against insulin resistance in male rats. The journals of gerontology. Ser A Biol Sci Med Sci (1998) 53:B19–24. doi: 10.1093/gerona/53A.1.B19 9467418

[16] Villareal DT HJ. Effect of DHEA on abdominal fat and insulin action in elderly women and men: a randomized controlled trial. JAMA (2004) 292:2243–8. doi: 10.1001/jama.292.18.2243 15536111

[17] Zhang X Xiao J Liu Q Ye Y Guo W Cui J. Low serum total testosterone is associated with non-alcoholic fatty liver disease in men but not in women with type 2 diabetes mellitus. Int J Endocrinol (2022) 2022:8509204. doi: 10.1155/2022/8509204 36065220 PMC9440833

[18] Zhang X Xiao J Liu T He Q Cui J Tang S. Low serum dehydroepiandrosterone and dehydroepiandrosterone sulfate are associated with coronary heart disease in men with type 2 diabetes mellitus. Front Endocrinol (Lausanne) (2022) 13:890029. doi: 10.3389/fendo.2022.890029 35832423 PMC9271610

[19] Zhang X Huang Y Xu N Feng W Qiao J Liu M. Low serum dehydroepiandrosterone levels are associated with diabetic retinopathy in patients with type 2 diabetes mellitus. J Diabetes Investig (2023) 14(5):675–85. doi: 10.1111/jdi.13997 PMC10119925 36811237

[20] Zhu D Society C. Guideline for the prevention and treatment of type 2 diabetes mellitus in China (2020 edition). Chin J Endocrinol Metab (2021) 37:311–98. doi: 10.3760/cma.j.cn311282-20210304-00142

[21] Joint Committee for Guideline R. 2018 Chinese guidelines for prevention and treatment of hypertension-A report of the revision committee of Chinese guidelines for prevention and treatment of hypertension. J Geriatr Cardiol (2019) 16:182–241. doi: 10.11909/j.issn.1671-5411.2019.03.014 31080465 PMC6500570

[22] Jun-Ren ZHU R-LG Shui-Ping ZHAO Guo-Ping LU Dong ZHAO Jian-Jun LI. 2016 Chinese guidelines for the management of dyslipidemia in adults. J Geriatr Cardiol (2018) 15:1–29. doi: 10.11909/j.issn.1671-5411.2018.01.011 29434622 PMC5803534

[23] Orentreich N Brind JL Rizer RL Vogelman JH. Age changes and sex differences in serum dehydroepiandrosterone sulfate concentrations throughout adulthood. J Clin Endocrinol Metab (1984) 59:551–5. doi: 10.1210/jcem-59-3-551 6235241

[24] Reiter EO Fuldauer VG Root AW. Secretion of the adrenal androgen, dehydroepiandrosterone sulfate, during normal infancy, childhood, and adolescence, in sick infants, and in children with endocrinologic abnormalities. J Pediatr (1977) 90:766–70. doi: 10.1016/S0022-3476(77)81244-4 140222

[25] Sklar CA Kaplan SL Grumbach MM. Evidence for dissociation between adrenarche and gonadarche: studies in patients with idiopathic precocious puberty, gonadal dysgenesis, isolated gonadotropin deficiency, and constitutionally delayed growth and adolescence. J Clin Endocrinol Metab (1980) 51:548–56. doi: 10.1210/jcem-51-3-548 6447708

[26] T. S-i Yamaguchi Y Yamakawa T Kimura M Ukawa K Yamada Y. Reduced serum dehydroepiandrosterone levels in diabetic patients with hyperinsulinaemia. Clin Endocrinol (2002) 52:727–33. doi: 10.1046/j.1365-2265.1998.00533.x 9861330

[27] Qin Y H OS Khani V Tan SC Zhi Y. Effects of dehydroepiandrosterone (DHEA) supplementation on the lipid profile: A systematic review and dose-response meta-analysis of randomized controlled trials. Nutr Metab Cardiovasc Dis (2020) 30:1465–75. doi: 10.1016/j.numecd.2020.05.015 32675010

[28] Kawano H Yasue H Kitagawa A Hirai N Yoshida T Soejima H. Dehydroepiandrosterone supplementation improves endothelial function and insulin sensitivity in men. J Clin Endocrinol Metab (2003) 88:3190–5. doi: 10.1210/jc.2002-021603 12843164

[29] Baulieu EE Thomas G Legrain S Lahlou N Roger M Debuire B. Dehydroepiandrosterone (DHEA), DHEA sulfate, and aging contribution of the DHEAge Study to a sociobiomedical issue. Proc Natl Acad Sci United States America (2000) 97:4279–84. doi: 10.1073/pnas.97.8.4279 PMC18228 10760294

[30] Gurnell EM Hunt PJ Curran SE Conway CL Pullenayegum EM Huppert FA. Long-term DHEA replacement in primary adrenal insufficiency: a randomized, controlled trial. J Clin Endocrinol Metab (2008) 93:400–9. doi: 10.1210/jc.2007-1134 PMC2729149 18000094

[31] Shufelt C Bretsky P Almeida CM Johnson BD Shaw LJ Azziz R. DHEA-S levels and cardiovascular disease mortality in postmenopausal women: results from the National Institutes of Health–National Heart, Lung, and Blood Institute (NHLBI)-sponsored Women's Ischemia Syndrome Evaluation (WISE). J Clin Endocrinol Metab (2010) 95:4985–92. doi: 10.1210/jc.2010-0143 PMC2968728 20739385

[32] Corona G Rastrelli G Giagulli VA Sila A Sforza A Forti G. Dehydroepiandrosterone supplementation in elderly men: a meta-analysis study of placebo-controlled trials. J Clin Endocrinol Metab (2013) 98:3615–26. doi: 10.1210/jc.2013-1358 23824417

[33] Elraiyah T Sonbol MB Wang Z Khairalseed T Asi N Undavalli C. Clinical review: The benefits and harms of systemic dehydroepiandrosterone (DHEA) in postmenopausal women with normal adrenal function: a systematic review and meta-analysis. J Clin Endocrinol Metab (2014) 99:3536–42. doi: 10.1210/jc.2014-2261 PMC5393492 25279571

[34] Jedrzejuk D Medras M Milewicz A Demissie M. Dehydroepiandrosterone replacement in healthy men with age-related decline of DHEA-S: effects on fat distribution, insulin sensitivity and lipid metabolism. Aging Male (2009) 6:151–6. doi: 10.1080/tam.6.3.151.156 14628495

[35] M. A Bednarek-Tupikowska G Kossowska B Bohdanowicz-Pawlak A Sciborski R. The influence of DHEA on serum lipids, insulin and sex hormone levels in rabbits with induced hypercholesterolemia. Off J Int Soc Gynecological Endocrinol (1995) 9(1):23–28. doi: 10.3109/09513599509160187 7793296

[36] L. EH Coleman DL Schwizer RW. Therapeutic effects of dehydroepiandrosterone (DHEA) in diabetic mice. Diabetes (1982) 31(9):830–3. doi: 10.2337/diabetes.31.9.830 6219024

[37] Kimura M Tanaka S Yamada Y Kiuchi Y Yamakawa T Sekihara H. Dehydroepiandrosterone decreases serum tumor necrosis factor-alpha and restores insulin sensitivity: independent effect from secondary weight reduction in genetically obese Zucker fatty rats. Endocrinology (1998) 139:3249–53. doi: 10.1210/endo.139.7.6118 9645700

[38] Aoki K Kikuchi T Mukasa K Ito S Nakajima A Satoh S. Dehydroepiandrosterone suppresses elevated hepatic glucose-6-phosphatase mRNA level in C57BL/KsJ-db/db mice: comparison with troglitazone. Endocrine J (2000) 47:799–804. doi: 10.1507/endocrj.47.799 11228057

[39] Campbell CSG Caperuto LC Hirata AE Araujo EP Velloso LA Saad MJ. The phosphatidylinositol/AKT/atypical PKC pathway is involved in the improved insulin sensitivity by DHEA in muscle and liver of rats in vivo. Life Sci (2004) 76:57–70. doi: 10.1016/j.lfs.2004.06.017 15501480

[40] Lin H-Y Yu IC Wang R-S Chen Y-T Liu N-C Altuwaijri S. Increased hepatic steatosis and insulin resistance in mice lacking hepatic androgen receptor. Hepatology (2008) 47:1924–35. doi: 10.1002/hep.22252 18449947

[41] Mauvais-Jarvis F Clegg DJ Hevener AL. The role of estrogens in control of energy balance and glucose homeostasis. Endocrine Rev (2013) 34:309–38. doi: 10.1210/er.2012-1055 PMC3660717 23460719

